# Home care nurses facilitating planned home deaths. A focused ethnography

**DOI:** 10.1186/s12904-023-01303-4

**Published:** 2023-11-09

**Authors:** Anne Kristine Sørstrøm, Mette Spliid Ludvigsen, Ingjerd Gåre Kymre

**Affiliations:** 1 Faculty of Nursing and Health Sciences, Nord University, Bodø, Norway; 2 Department of Clinical Medicine - Randers Regional Hospital, Aarhus University, Aarhus, Denmark

**Keywords:** Home care nurses, Home care services, Palliative care, Terminal care, End of life care

## Abstract

**Background:**

Home care nurses provide complex palliative care for patients who want to die in their own homes. This study aimed to explore home care nurses’ facilitation of planned home death to better understand nursing practices.

**Methods:**

Data were collected between March 2019 and March 2020 using participant observations and semi-structured interviews. In addition, the number of planned home deaths was recorded. The analysis was guided by Roper and Shapira`s framework on focused ethnography.

**Results:**

Twenty home care nurses (three men) in eight home care areas in two Norwegian municipalities met the inclusion criteria. Eight home deaths were registered, seven participatory observations were performed, and 20 semi-structured interviews were completed. Home care nurses find facilitating planned home deaths to be rewarding work, to the point of going above and beyond. At the same time, they describe facilitating planned home deaths as demanding work due to organizational stressors such as staff shortages, heavy workloads, and time restraints. While they tend to patients’ needs, they also express concern for the wellbeing of the next of kin. They find it challenging to juggle the needs of the patients with the needs of next of kin, as these are not always correlated.

**Conclusion:**

Home care nurses are pushing the boundaries of their practice when facilitating planned home deaths while compensating for a fragile system by going above and beyond for patients and their next of kin. Providing insights into the work of home care nurses providing palliative care in patients’ homes can impact recruiting and retaining nurses in the workforce and influence local practices and policies.

**Supplementary Information:**

The online version contains supplementary material available at 10.1186/s12904-023-01303-4.

Where people die is an important determinant of the patient and caregiver experience [1]. Most people would prefer to die at home [2]. Sociodemographic factors can influence the place of death, such as marital status, family support, and caregiver preferences as well as living arrangements, urban and rural settings, and predictable disease courses [3]. However, a planned home death (PHD) can be challenging for the dying person and family members. Home care nurses (HCNs) have a role in caring for terminally ill patients and may be confronted by extraordinary tasks and rather private and sensitive situations in peoples’ private homes. Although it is not new for nurses to provide nursing care in people’s private homes, as more people want to die at home more HCNs will experience these new and unfamiliar care situations [1]. A better understanding of how HCNs experience facilitating PHD may help develop policies and services that ensure high-quality care at the end of life. We assume that insights from such research will impact both policymaking and local routines, and thus we call for further exploration of HCNs’ facilitating for PHD.

## Background

Palliative care has been recognized by the World Health Assembly resolution as an ethical responsibility of health systems, prompting World Health Organization (WHO) member states to commit to ensuring high-quality palliative care through primary care services [4]. The WHO defines palliative care as ‘*an approach that improves the quality of life of patients and their families facing the problems associated with life-threatening illness, through the prevention and relief of suffering by means of early identification and impeccable assessment and treatment of pain and other problems, physical, psychosocial and spiritual*.’^p1^.

Palliative care in the home intends to provide high-quality holistic care in order to tend to patients’ physical and psychosocial needs in a home setting [5]. The principles of palliative care and palliative care at home remain consistent even though service provision has undergone changes due to the reorganization of the healthcare models and advances in symptom management and treatments [6].

The need for competent and accessible high-quality palliative care is pressing and requires strengthening of the general palliative care services [7]. Currently, community practices face many challenges such as aging populations, decentralization of health care, shortages of nurses, and an increasing number of patients are dying from complex diseases, including dementia, cancer, and other chronic diseases [8]. Research indicates that most patients in a palliative setting want to spend as much time as possible at home, and many want to die there [9, 10].

Palliative care is complex and covers a wide array of physical, psychosocial, and existential challenges. Home care nurses (HCNs) face challenges that specialist care services do not, i.e., geographical distances and poorly suited facilities [8]. Planned home deaths (PHDs) are associated with the provision of home care nursing [11], and providing high-quality palliative care for patients in their homes at the end of life is a part of the job description for HCNs in many high-income countries, as they are often described as lynchpins of community palliative care [12].

Norway has one of the lowest rates of PHDs of the Western countries [11], and Statistics Norway reports that 13% of the population die at home, but this percentage includes suicides, accidents, and unplanned deaths due to frailty of health [13]. The number of actual PHDs is unknown, but recent research indicates that the rate of potential PHDs could be less than half of the registered home deaths, or approximately 7% [11].

HCNs have a significant role in caring for patients in need of palliative care and in the facilitation of PHD, meaning the organizing of the care and nursing needed when patients express a wish to die in their own home [14]. However, the literature points out several issues affecting HCNs’ work with patients dying at home, including a lack of both knowledge and skills in palliative care and a lack of support for both informal carers and healthcare professionals [15, 16]. Other examples of issues include long travel distances and nursing staff shortages [17–19] and the availability of local healthcare services [20]. Service shortfalls and lack of clinical expertise exist in regional and rural areas in many countries [21].

Earlier studies from the UK identified the HCN’s pivotal role in providing palliative care and facilitating PHD [22, 23]. A review of barriers to palliative care at home in rural areas published in 2009 reported a range of negative factors, such as geographic isolation, infrequent cases of palliative care patients, and blurred work boundaries [24]. However, previous studies have become outdated due to the developing and changing role of HCNs and to reforms in home care services [25]. Only a few studies have examined nursing practices to facilitate PHD. Moreover, the attitudes of HCNs caring for the dying have received little attention prior to 2010, as has consideration of how their attitudes affect nursing interventions and the facilitation of patient preferences at the end of life [26].

Based on this literature, we note the paucity of knowledge related to the topic. Moreover, evidence suggests that there is a need for more research focused on observations of real-life home care nursing practice, more precisely, what HCNs *do* in practice. This study aimed to explore HCNs’ experiences and practices of caring for a dying person at home in order to better understand their experiences in facilitating PHDs.

## Methods

The facilitation of a PHD is a rather distinct study phenomenon within the home care nursing setting. The ethnographic objective of unveiling the shared aspects of culture, including values, beliefs, knowledge, skills, and actions, is well-suited to this context [27]. Using a wider range of research methods, specifically observational methods, enables a more detailed investigation of HCNs’ clinical practice [28], and we considered Roper and Shapira’s qualitative framework on focused ethnography design to be suitable [29]. This design enabled us to explore HCNs’ bedside practice with a focus on the facilitation of PHD in these private and sensitive situations [28]. The fieldwork involved short-term and targeted data collection sessions with participant observations and individual interviews with HCNs. Thus the participant observations and individual interviews with carefully selected participants could be structured around the study topic [30]. The first author and co-authors strived for flexibility, adaptability, and rigor in the data collection [27]. We employed a data triangulation of participant observations, semi-structured interviews, and analysis of the registrations forms to gather rich data and thus better understand the complex experiences of PHD from the HCNs` perspective. This resulted in valuable insights not generally accessible through standard research methods such as solely retrospective qualitative accounts.

### Setting

The study setting was eight different home care services in two municipalities (50,000–80,000 inhabitants) consisting of both rural and urban areas in northern Norway. Norway is a vast country, with rural areas having sparse and intermittent home care services and long travel distances. The Norwegian healthcare system is based on the principles of public welfare and provides free and universal healthcare to all. Municipalities are required to provide free home nursing services to their inhabitants [31], including basic palliative care integrated into general patient care [32]. National policies and government health initiatives have put an increased emphasis on providing palliative care in primary health care closer to the patient`s home [7]. These changes can be attributed to the introduction of the Norwegian Coordination Reform (2012) [33]. This reform has placed a considerable responsibility on the municipalities as they now play a prominent part in meeting the growing demand for health services [33]. Within the municipalities, HCNs provide home care nursing comparable to the role of ‘community nurses’ in Australia and Canada and ‘district nurses’ in the UK [18]. For clarity and consistency, this study will use the term ‘home care nurse’ (HCN).

### Participants

HCNs employed in home care areas and who had experience with facilitating PHD were eligible for inclusion. We used purposive sampling to recruit HCNs from the home care services in order to ensure specific knowledge and experience in facilitating PHDs. In co-operation with the nurse mangers in the home care service, the first author identified eligible HCNs that met the inclusion criteria. They were contacted by the managers inviting them to participate, and those who expressed interest in participating were approached by the first author with information and a formal request to participate in the study. Included HCNs were asked if they knew other HCNs with experience in facilitating PHD, thus acting as referral agents. Potential cases of PHDs to observe were identified by an ambulatory cancer nurse who was a knowledgeable insider and who acted as a gatekeeper when mapping appropriate cases for participatory observations. She informed the patients and next of kin (NOK) about the project and helped to establish whether the researcher`s presence was acceptable.

### Data collection

Data collection took place from March 2019 to March 2020. In accordance with the ethnographic approach with the goal to study how HCNs think and act in their natural setting, we used individual semi-structured interviews and participant observations. In addition, we registered the number of PHDs in the whole municipality during the period.

### Participant observation

The first author observed HCNs when they facilitated PHDs for dying patients. During the participant observations, we gained deeper insights into what HCNs do when caring for dying persons and their NOK. Each observation session included the time HCNs spent preparing for the visits at HCN offices, time spent traveling to and from patient’s homes, and time spent during visits. Due to the sensitive nature of the topic being investigated, the researcher did not take notes in the patient’s homes. However, to avoid memory bias observations, reflective notes, and verbatim quotes were noted immediately after home visits. These were written in accordance with an observation guide inspired by Spradley (1980) [34]. The guide acted as a reminder of what to pay attention to during the observations, such as the physical place, the people involved, the norms of the group, the activities they carried out, and the emotions they felt and expressed. During the observations in the patient’s home, the researcher sat down with the HCN, patient, and NOK when invited. When HCNs carried out clinical tasks, such as managing analgesics or changing a urinary catheter, the researcher adopted an observer role without being invasive or obtrusive. The purpose was to observe and understand from the view of the participants rather than add personal interpretations and explanations.

### Interviews

Interviews are important in focused ethnography due to the constricted time frame of fieldwork [35]. We employed semi-structured interviews with a set agenda, yet with the flexibility to pursue the expressed ideas of the participants [36]. The interviews were used to collect data on what we could not or had not observed, such as attitudes and emotions. The interviews helped validate the observations and thus provided directions for future observations, as well as observations giving directions for subjects requiring further explanation in the interviews. The interview guide (additional file A) was developed for this study by the first author in collaboration with the co-authors based on the literature review and the study aims. The guide included open questions such as “*Can you describe how you facilitate a planned home death*” and “*What challenges do you face when facilitating planned home deaths*”. When appropriate, follow-up questions were asked, moving from the general to the more specific by probing (e.g., *“could you tell me more about that”* or “*what do you mean when you say that*”. The interviews took place at a suitable location at the HCNs’ places of work. The interviews took an average of 50 min and were audio recorded and transcribed verbatim. The interview guide was fine-tuned over the course of the study as observations revealed issues we wanted to explore further. The interviews as a whole sought to encourage participants to articulate their experiences, beliefs, practices and attitudes in order to identify practice patterns that are characteristic of this sub-culture through active listening and attention to non-verbal signs and by showing empathy [34]. Although the participants were offered the opportunity to read the transcribed material, they all declined.

### Registrations of the number of home deaths

The registration form was reviewed and accepted for use by a knowledgeable insider. The documentation was completed by an ambulatory cancer nurse employed by the home care service, responsible for monitoring all PHDs within the municipality throughout the data collection year. The information gathered from these records was utilized to determine the count of PHDs that were successfully provided by the home care services, along with the tally of requested PHDs that were not fulfilled, along with an explanation for the unmet requests. If the patient did not die at home, even though it was an expressed wish, the alternatives for boxes to check why included insufficient personnel, lack of competence, lack of equipment, changes in the patient’s wishes, the NOK`s situation, the need for advanced symptom management, and others. No identifying patient information was registered.

### Data analysis

Field notes and transcribed interviews were read and reread to get an overview of the data. The transcribed interviews were analyzed first, followed by a separate analysis of the field notes. Then all the data, including data from the registration forms, were analyzed together to identify similarities or discrepancies. We used NVivo 11 (QRS International, 2017) to organize the data. The initial analysis was conducted by the first author and discussed and developed in collaboration with the co-authors. Roper and Shapira`s framework (2000) was employed using the following five steps: coding for descriptive labels, sorting to identify patterns, identifying outliers, generalizing constructs and theories, and taking notes of reflections and insights made by the researcher [29].

This is a circular process, not linear or chronological. However, to describe the process we present it here as continuous steps. Answering the study`s aim, related and similar segments of text were grouped and coded to form meaningful categories we called descriptive labels. The length of the data segments in the descriptive labels were reduced in size without losing content in order to form manageable units. We used in vivo coding when applicable. We started with broad codes, such as ‘*next of kin*’, that were later broken into smaller concepts, i.e., ‘*feeling of concern for next of kin*’. We clustered these labels in order to identify patterns and themes, visually separating them into general and abstract themes that could explain relationships in the cultural setting of the home care services. We then rearranged the labels in order to identify patterns, such as similarities or differences in how HCNs performed or reflected on PHD, in their cultural setting. These similar patterns formed categories, which were of manageable sizes. From the sorted categories, we identified themes and drew connections between the emic perspectives of the participants and the etic interpretations of the researchers in order to construct theoretical understandings that included both perspectives. We formulated and defined overall themes that included relevant categories. Themes are defined as larger sociological categories, group behaviors, shared opinions, etc. [37]. These were defined again and compared with compatible existing constructs, theories, literature, and research in line with the analytical framework [29].

In every step of the analysis we were aware of the potential for outliers, namely statements or actions that do not ‘fit’ with the rest of the data [29]. We identified two statements as outliers from the interviews. These were followed up and examined to see if they were extraordinary responses, i.e., true outliers, or if they were common traits shared with other HCNs.

Note-taking was done continuously throughout all phases of the data collection and the analysis to record first impressions, identify preconceived notions, and support the audit trail. The credibility of the coding, categories, and identification of the outliers was checked for accuracy by the team of authors.

We translated the findings and quotations from Norwegian into English. We strived to correctly translate the participants’ expressed views and descriptions. Verbatim quotations are used to exemplify the themes and represent the participants` understanding as accurately as possible in order to give a better understanding than the researcher’s paraphrasing could.

### Ethical considerations

Because the researcher could inadvertently gain information regarding other patients not relevant to the study when observing HCNs’ work, an exemption from the duty of confidentiality was obtained from Regional Committee for Medical and Health Research Ethics in Northern Norway (REC) (2019/605). According to Norwegian law, no further ethical approval was required. All collected data were anonymized and kept secure and confidential at all times. This study was performed according to Norwegian Centre for Research Data (NSD) (77,356) regulations (now known as the Norwegian Agency for Shared Services in Education and Research). The HCNs were informed verbally and were provided with a participant information sheet and gave their written informed consent to participate in the interviews and observations. All patients and NOKs who were approached agreed to participate in the study. All patients and NOKs were informed about the study, and written consent was collected that permitted the researcher to observe the work of HCNs in their homes.

We were unable to gain knowledge about HCN’s facilitation of home death without close contact with dying patients and their NOK. Therefore sensitive issues and ethical concerns were carefully considered. We reflected on the potential to intrude on an intimate and private experience and how to address any distress we may inadvertently cause. Guided by Sivell and colleagues (2019), we used sensitive and open questioning, researcher self-disclosure, awareness of power relationships, and adaptation to the individual needs of the patients on a case-by-case basis [38].

### Findings

Twenty-one HCNs were approached with requests to participate in the study, and one declined participation without providing a reason. Twenty HCNs were interviewed, 17 of whom were women, and the HCNs were 25–63 (mean 42) years old with 1–24 (mean 12) years of experience. Their experience in facilitating PHDs ranged from two cases to more than ten cases (Table 1). Three of these were also observed when they were facilitating PHDs. They were selected because they were on duty when the patients had scheduled visits. Of the nine home care service areas contacted, one did not have any HCNs that met the inclusion criteria at the time of study recruitment. One interview was interrupted after 30 min due to a work-related emergency.

The researcher followed three different HCNs facilitating PHD for two dying patients and their NOK. This amounted to seven different observations, including four with the HCN, NOK, and the patient, two with the HCN and NOK, and one with just the HCN. These observations included visits to the patients’ homes, bereavement conversations, and one session with an HCN in a car, observing her getting supplies for a PHD and making phone calls about the PHD. From the registration forms, we counted eight patients wanting to die in their own homes. Five of those died at home. The reasons for not dying at home were in all three cases related to the NOK’s situation. In total, participatory observations amounted to 8.5 h. The researcher took on different roles during the observations. At times, like during medical interventions, she assumed the role of a silent observer when the HCNs were focused on clinical tasks. On other occasions, it was natural for her to engage in conversations with the patient and/or their NOK, for example, when they offered coffee and biscuits.

**Table 1:** Characteristics of the study participants

Characteristics	Study participants n = 20
Sex, n	
Women	17
Men	3
Age, mean (range)	43 (23–64)
Experience as a nurse, years (range)	12 (1–24)
Specialization among nurses, n	
Palliative care	2
Adult psychiatry and substance abuse	1
Dementia and geriatric psychiatry	2
Cancer care	8
None	7
Experienced cases of facilitating planned home deaths, mean (range)	5.9 (2–10)

Data presented as numbers or means (range)

The analytical process led to the identification of three main themes that characterized HCNs’ experiences of facilitating PHDs, namely going above and beyond, juggling the patient’s and NOK’s needs, and demanding work We present them here separately but recognize that the themes are interrelated and overlapping. HCNs find the work of facilitating PHDs rewarding, and it is important to them to guarantee availability and flexibility for patients and NOK. However, they reported challenges when facilitating PHDs in the forms of staff shortages and high workloads. In addition, they expressed concern for the well-being of NOK as they juggle the needs of patients and the needs of NOKs. The three themes are presented in the following sections.

### Going above and beyond

HCNs in this study clearly expressed positive attitudes about facilitating PHDs and emphasized that this was rewarding work. The HCNs commented on the value of facilitating PHDs numerous times, and they used words like *rewarding*, *meaningful*, and *purposeful* and said that they *took pride in it*. One nurse said:

‘*You are alone most of the time in the home care service. I found it really scary the first time. But at the same time it was nice to see…it was her greatest and last wish, to die at home. It was really nice making that happen for her. Even though it was hard for me, it was fulfilling to see that we made her wish come true.*’ (HCN 18).

HCNs stressed how they did everything within their power to give patients and NOK the best possible care. Observations also revealed how HCNs repeatedly told patients and NOK to call the HCN if they required assistance without specifying which tasks they referred to. An HCN explained it like this in an interview:

*‘We don’t usually give away the on-call number, but in the case of PHD we give it to both the patient and NOK so they can easily contact us, whenever and for whatever reason.’* (HCN11).

The HCNs expressed going above and beyond, and some of the HCNs from the more rural home care areas demonstrated a collective drive or an ethical duty to provide PHD as an option by using phrases like ‘*We do everything in our power*’ and ‘*We want it to happen’*. One HCN stated in the car after a house call:

*‘Like I said, we do everything we can and more to make this happen.’ (Observation 5)*.

Several HCNs stated that they would go far to aid patients and NOK, and some of them mentioned how they had rescheduled or cancelled their planned vacations to ensure nurse coverage during a summer when a patient in their home care area wanted to die at home. Another HCN explained how she had put a folding cot in her office so she could spend the night there and thus have a shorter drive in case the dying patient and their NOK needed anything. This was the first of the two outliers identified in the data and was used to confirm the notion of HCNs going out of their way to accommodate a patient’s wish to die at home.

On a field visit to a dying patient’s home one Monday, the patient and NOK explained to the HCN that the patient had been briefly admitted to an institution during the weekend due to exacerbation of their cancer pain. The HCN asked why they had not called her during the weekend. The spouse of the patient replied that they knew she was at her cabin, given she had the weekend off. The HCN then said: ‘*You should have called me anyway. I am always available for you.*’ *(Observation 1)*.

Another HCN commented in the interview:

*‘We go above and beyond. Every time. We want it to happen as much as they do. After all, it is somebody else`s life, or in fact somebody else’s death. You only get one chance.’ (HCN12)*.

### Juggling the patient’s and NOK’s needs

The dynamics between the NOK, the patient, and the HCNs emerged as an important issue due to conflicting needs. According to HCNs, NOK are often in danger of being exhausted, worn out, and scared. Their needs were highlighted as equally important as the patients’ needs but more challenging to care for. Their needs were described as not homogenous, nor was the way they coped with the situation. When asked ‘*What do you consider important when facilitating for planned home deaths*’, one HCN replied:

*‘We don’t have enough focus on family rotations. They don’t get it. I don’t think it is explained in detail. The family has to work shifts to make it happen.’ (HCN2)*.

The HCNs in our study pointed out how on several occasions the NOK were an essential factor in facilitating PHDs and how PHDs could not be facilitated without them. There were similar perceptions among the HCNs that PHDs were not possible without NOKs being present and willing to take on the majority of the day-to-day care.

'What I see is the next of kin. A lot of weight is put on them. It’s useless if…Honestly, it is not enough with just a spouse.'

*(HCN 13)*

The participants expressed that facilitating PHDs for patients who had young children was a challenge. To see their emotional struggles took a toll on the HCNs, and several of them reflected on such cases:

‘*It ended up being challenging for the children…the last couple of days. That’s why XX went to the hospital and died there. It can seem ok in the beginning. Like, ok, we’re all together as a family…but, I think it gets… The children cried a lot and didn’t want to go to school, and it just got too complicated at home. I would not do that again.’ (HCN2)*.

Much of the work the HCNs did was organized around the medical needs of the patients, but they focused a lot of attention on the NOK. Meeting their needs and listening to their worries and comforting them were sometimes the biggest issues. HCNs normally plan their shift according to a worklist; a detailed list of patients to visit, their care needs and estimated time usage. However, visits to PHDs were not timed like other visits, and the HCNs seemed to exhibit more flexibility and discretion in time management when visiting dying patients. The HCNs never looked at their phones or appeared to be busy or rushed when meeting with patients or NOKs. However, after leaving the dying patient and NOK, the HCNs explained that they were in a hurry, and now had other patients waiting for them.

NOK were perceived as essential when facilitating PHD and as making a substantial contribution to the care of the dying. However, barriers to PHDs lie in the workload put on NOK when patients die at home. The NOK could be so exhausted that they do not feel safe with the patients at home, even though it is the expressed wishes of the patient.

*‘Quite often it feels like what you do is primarily for the next of kin, but that’s just the way it is.’ (HCN3)*.

Two different HCNs both described a fear of NOK getting flashbacks after a period of palliative care in the home:

‘*I find next of kin the most challenging factor. Because they are so different, some are fine with things and handle it well, but others don’t. They are so important in making this happen, and we must make sure that we don’t get it wrong, and they end up having terrible flashbacks later on.*’ *(HCN11)*.

*‘I think I would say that next of kin are a barrier for planned home deaths. They are so important, and you must make sure that it does not get overwhelming. That they feel that they have to do this because it is expected and then it backfires later, and they get painful flashbacks.’ (HCN9)*.

This can imply that HCNs work within a tension that exists in the home, where different needs are not being met. The following excerpt from the field notes made on a visit to a dying patient and NOK further illustrates this point:

‘*The nurse (HCN6) asks the patient if more frequent visits from the HCNs could be an option, particularly with regards to assistance when showering. The patient replies (annoyed?) it is not necessary yet. That they don’t want more people coming into their home. The spouse stares down in the table. The nurse looks closely at her and asks directly but gently, “Do you need some assistance, some relief?” The spouse tears up but does not look up*.’

*(Observation 2)*

These excerpts demonstrate some of the challenges faced by HCNs when trying to balance aiding the NOK and respecting the patient’s wishes, all while clinically assessing the patient’s needs. None of the HCNs performed a systematic assessment of the NOK’s needs or wellbeing, but all HCNs expressed a concern for their unmet needs. The HCNs were attentive and supportive by listening to their grievances. HCNs were observed trying to provide reassurance and to console the NOK before, during, and after the death of their loved ones. As seen in the field notes, some NOK were unable to reconcile with reneging on the promise of caring at home until death, even though the situation demanded a hospital admission, as the following demonstrates:

*‘During a bereavement follow up, the HCN (HCN4) asks the widow if she is troubled by a bad conscience for the patient dying in xxx (an institution). The widow whispers: “It tears me apart”. The nurse replies: “But you were so tired and worried in the end. You couldn’t have done it!”…. “I should have stuck it out”, the widow replies.’ (Observation 7)*.

The HCNs explained how the patient’s spouse or NOK often needed a space to talk freely without the patient being present. These talks often took place on the porch when they escorted the HCNs out of the home after visits. As described in the fieldnotes:

*‘The spouse leads us out in the hallway while the patient remains in the living room as we say goodbye and get our coats on. She follows us outside on the steps in the cold weather and tells us quietly, almost whispers that she does not get any breaks and that she is very, very tired. She starts crying silently, clearly not wanting the patient to hear or see her like this. The nurse pats her on the back, asking if there are any other relatives that can be with the patient while she gets a break.’ (Observation 3)*.

### Demanding work

All HCNs stated that even though the work was rewarding, it took its toll on them, both emotionally and physically. The reasons for this were blurred work boundaries, and they all repeated numerous times that they would go a long way to support patients and their NOK. They described their work as being a game of solitaire, where the challenge was to make it all add up because even though they had an ongoing PHD they still had 300 other patients requiring visits and care. They explained how PHDs require extensive coordination as well as correspondence with other professions and institutions such as the general physician, the palliative team at the local hospital, and the pharmacy. Most of these calls and follow ups were performed in the car on their way to and from patients’ homes and between other visits. Some HCNs reported personal health challenges related to their work, and four HCNs stated in interviews that they have had periods of sick leave as a direct consequence of facilitating PHDs that were especially strenuous and time-consuming. They talked about other HCNs making an advent calendar for dying patients’ children and questioning where the limits to their jobs were supposed to be drawn, and who was responsible for drawing those lines. HCNs reported that the patients and NOK are satisfied with, and even grateful for, the care provided. However, four of the HCNs in this study asked themselves, ‘*At what cost?*’ (HCN3, HCN7, HCN15, HCN17).

*‘It is a horrible feeling. Doing everything you can, but it is never enough.’ (HCN7)*.

All HCNs in this study stated that there were not enough nurses in the workforce, and they called for more qualified and knowledgeable staff. The shortage hindered them from doing a good job and kept them from suggesting to eligible patients that dying at home might be an option for them. They slot palliative patients in where they can, while at the same time making sure the dying patient and NOK experience prioritization, availability, and flexibility by, for example, not registering time usage during visits. Many of them spoke of the constant stressor of being understaffed and having a huge workload, and this became worse when there were PHDs to facilitate. One HCN stated:

‘*We are stretched pretty thin as is.’ (HCN4)*.

They talked at length about the frustrations of having to manage the care of patients on their worklist while being flexible and accessible to dying patients and their NOK’s individual needs. To fulfill these requirements within a given timeframe was reported as challenging, and the understaffing in combination with the high workload created a void where they felt they made compromises on the quality of care. They implied that continuity of care could only be achieved when every patient received the right kind of care at the right time.

HCN8: ‘*There have been times where we advised against PHDs because we didn’t have the required competence and we didn’t have enough nurses to the extent that we had to be honest and say, we want to but it is not possible…’*.

Interviewer: ‘*Is that hard? Saying no to patients?’*

HCN8: “*Yes, very hard. Very hard. We took it personally, and it felt like a failure in that good palliative notion, that good palliative environment and attitude, the hospice philosophy we are trained in, and our core values. But we had to restrict ourselves and analyze the situation, and if you disagree and have objections, take it up with the person in charge.”*

Another HCN put it like this:

*‘We have become extremely mentally worn out.’ (HCN9)*.

One HCN in an urban area was skeptical to the notion of facilitating PHDs and stated in the interview that they did not have any PHDs in her area in recent years. She explained how it would not be justifiable due to the lack of nurses. This was identified as the second outlier in that it did not fit with the patterns identified in the rest of the data.

Evidently, HCNs’ feelings for PHD were both positive and negative. Most HCNs expressed a solid commitment to the patient’s and NOK`s needs. However, many of the difficulties experienced by the HCNs were described as organizational challenges. They explained how municipalities have failed to prioritize home care to the extent necessary to ensure adequate nursing coverage and how additional qualified staff and a reduction in workload could provide coordinated, continuous, and appropriate care.

## Discussion

This study gives insight into HCNs’ experiences in facilitating PHDs. We registered five PHDs in a Norwegian municipality during the course of a year. From participant observations, fieldnotes, and qualitative interviews we developed three themes regarding HCNs’ experiences when facilitating PHDs. We found that HCNs – both as individuals and collectively – experience facilitating PHDs as rewarding work. They welcome more PHDs as they find this work to be important, but do this by *going above and beyond.* They also find facilitating PHDs to be challenging and describe it as *demanding work* due to time constraints, high workloads, and staff shortages. They also express a concern for the wellbeing of the NOK *when juggling their needs with the needs of patients.*

### Going above and beyond

The HCNs find facilitating PHDs to be rewarding work and want to offer eligible patients the opportunity to die at home if they want to. Similar findings have been found in other studies, describing it as work that generates positive feelings and work that gives a lot back to the HCNs [39]. Studies point out how HCNs have personal commitments to patients receiving palliative care [40] and how an actual desire to do good influences their approach to care [12]. The HCNs’ perception of PHD as a rewarding practice is visible through their demonstration of extreme flexibility that shows that they will go far in making sure that the dying patient dies in their preferred place. As such, our findings confirm previous research that describes similar compensation mechanisms [40]. One issue of importance that can make HCNs reluctant to suggest PHD as a viable option is experiences of staff shortages and time constraints. The low number of registered home deaths in our study could be explained by the proximity to a regional hospital in an urban area. Contact with hospitals is related to hospital death, while PHDs are more likely to happen in rural areas, despite challenges in accessing health care and palliative care [20]. In Kjellstadslie´s study, municipalities with fewer inhabitants were associated with more PHDs [11]. This could be describing a common societal experience for the home care area, and might imply a high degree of collective commitment to providing a PHD when it is the explicit and expressed wish of the patient.

This indicates a personal and cultural compensation for organizational shortcomings, and in fact these shortcomings are preventing HCNs from providing equal care to all patients and making them choose which patients to prioritize. However, this display of extreme flexibility in combination with an increase in workload, tasks, and responsibilities over time [12, 41, 42] can indicate a negative effect on the nature of the work and can lead to blurred work boundaries.

### Demanding work

The HCNs in our study experienced a high workload and limited amount of time to perform their patient-oriented tasks in the home care services. Palliative care at home is time consuming and requires spending time with patients and NOK. Adding a PHD to a generalist caseload is perceived as challenging and exhausting by the HCNs. This is in line with previous studies reporting that time constraints are a factor of considerable stress for HCNs [39, 43]. They often prioritize care for palliative patients, resulting in other patients having to wait [39]. These findings suggest that HCNs want to provide optimal, person-centered care to the dying patients and their NOK, but this is at the expense of time available to other patients. This in turn makes the workload and time pressure build up. In general, HCNs spoke of a service with sparse resources and of palliative care patients on top of full work lists with other patients having complex needs in addition to long travel distances. Other studies have also described the combination of travel distances and high caseloads as a cause of stress for HCNs [39, 44]. Few measures have been taken to ensure adequate staffing and competence to serve the growing and complex patient group that the home care service tailors to [45]. Consequently, the HCNs’ workloads have increased with more patients with complex needs, generating increased administration and documentation and thus leaving less time for patient-oriented tasks.

Over time there has been a prominent growth in the municipal community health services in Norway. The services now have to adapt to new and challenging tasks, while the supply of HCNs and competence development has not been able to keep up with these increased demands [46]. The way the services are organized will affect the quality of the care. According to Gautun, Oyen, and Bratt [45], staff shortages escalate with increasing municipality size. Previous research has reported a lack of resources, i.e. personnel and equipment, to take on the challenges in the wake of implementing the Coordination Reform and specifically points to a need for more qualified personnel [47]. More HCNs in the work force and on shifts can reduce workloads, increase quality standards, and contribute to a larger professional environment. The responsibility to make challenging prioritizations should not be put on the individual HCN [46]. There is a clear and significant connection between the lack of nurses and quality of care, as well as between the lack of nurses and professional commitment [45]. Insufficient staffing levels might lead HCNs to subtly dissuade families from considering a PHD or refrain from mentioning it as a viable option. This could result in underreporting the actual number of patients desiring a PHD and act as a barrier to providing comprehensive palliative care.

### Juggling needs

In general, the HCNs in our study stressed the importance of emotional support for the NOK as well as the patient. They reported that NOK were essential in achieving time at home for the patient. This is supported by previous studies stating how care for the patient’s family is a particularly important part of the nurse’s role in palliative care [48]. It has been reported that the feasibility of PHD heavily relies on the involvement of NOK to make it possible [49]. However, studies specify that assessing needs is typically focused on the patient rather than the NOK [42]. NOK often find themselves managing complex physical and emotional issues and thus do not attend to their own needs, only those of the patient [50]. The findings in our study indicate that the HCNs struggle with juggling the needs of the NOK at the same time as tending to the patients’ needs and wishes because their needs can sometimes be contradictory. HCNs in our study were concerned for the wellbeing of NOK in terms of getting it right, not having painful flashbacks, and not getting worn out, which have been previously reported [42]. A British study calculated the amount of time NOK spend on patients with advanced cancer as 69 h per week in the final 3 months of life [14]. Neergard points out the importance of HCNs helping NOK maintain a balance between their needs, wishes, and resources [51]. None of the HCNs in our study stated that they used a systematic approach to identify the needs of NOK. They were, however, observed doing so in a more informal way, such as over a cup of coffee or on the patio when the NOK escorted the HCN out, and often on the initiative of the NOK. This phenomenon has also been described by Becqué, where family caregiver support is primarily given at the end of visits, on the doorstep while leaving [42]. This can result in arbitrary assessments and assumptions about the NOK’s needs and wellbeing, as well as said needs being unmet [42]. To assess NOK’s capacity – meaning their ability and willingness to provide informal care to dying patients – is important [19]. This could be assessed by collecting information about factors such as age, health, experience, availability, and emotional state and if they attend to their own needs [19]. NOK do care for the patient and take care of their needs, but some get to a point where they cannot take it anymore and can no longer cope [52]. This confirms the findings in our study, where three patients who wanted to die at home were admitted to an institution due to the NOK’s situation.

HCNs can be unaware of the unmet needs of NOK [53], although the findings in our study suggest that the HCNs are aware of their needs, but there are limits to what the HCNs can do to accommodate such needs. Our findings show that they do spend time building a close and reassuring relationship with the NOK, by, for example, providing them with a phone number that provides 24/7 availability. They make suggestions to patients and NOK to get more help and assistance when the HCNs see a need for it. Similar negotiations have been identified where HCNs actively mediate with NOK regarding the level of service needed in the home [8].

A study from 2016 showed that patients who wanted to die at the hospital did so on the premise of feelings around protecting NOK from physical and emotional burdens if they were to stay at home, as well as wanting the safety of clustered expertise in the specialist palliative care unit [54]. Challenges they feared could arise at home included difficulty breathing, difficulty in accessing necessary symptom control, and “wrecking” the place where NOK will continue to reside after the patient’s death [54]. These results can be related to our findings, where HCNs expressed a notable fear of NOK having too much responsibility and carrying too heavy a load. Similar findings in other studies [55, 56] have been described as a frustration and lack of preparedness for the extent of care necessary. Studies have shown how NOKs are prone to emotional, financial, and physical distress when caring for their dying family member [42, 57]. HCNs in our study explained a fear of bereaved NOK having painful ‘*flashbacks*’ and being left with unprocessed trauma, correlating to ‘wrecking’ the place NOK will inhabit after a loved one has passed. The concept of the home as a place of death is multifaceted. According to Milligan et al. (2016) NOK may find a sense of fulfillment in “doing the right thing,“ however the experience of a death at home can disrupt their feelings of home, belonging, and identity [58]. A meta-ethnography from 2018 reviewed several studies reporting a key barrier to patients achieving death at home as the burden placed on both informal caregivers and families. Informal caregivers frequently reported experiencing physical and emotional exhaustion due to the responsibility of providing care to their loved ones, particularly when dealing with elderly or isolated patients [16]. One study underscored the significance of acknowledging that informal caregivers are not only providers of care but also recipients, necessitating substantial attention to their needs alongside those of the terminally ill patient [59].

Several HCNs in this study brought up cases where young children were involved as a particularly challenging issue related to PHD. This confirms what other studies have reported, that dealing with children is a top source of stress for HCNs [60, 61]. In Dunne’s study, HCNs expressed that dealing with children and adolescents in the family of a dying patient was emotionally challenging. This, in turn, contributed to the overall stress of providing palliative care in the home [61]. To see family members suffer takes an emotional toll on the HCNs [39]. Dunne’s study points out how HNCs felt inadequate and helpless when meeting children and adolescents and as a consequence excluded them from involvement and care [61]. Nurses in one study highlighted barriers for supporting children as next of kin: many of them lack the necessary skills, qualifications, and experience to effectively support the child. This deficiency results in hesitancy to communicate and engage with the child. The resulting uncertainty fosters a desire to avoid such situations altogether [62]. However, other studies have shown how children as NOK want to stay at home with dying parents. This allowed them to monitor changes in the illness, fostering a sense of security and helping them cope with anxiety and grief. Being caregivers also created opportunities for making happy memories and having meaningful conversations. Many preferred their parent not to die in the hospital, honoring their wishes and avoiding the hospital’s intimidating atmosphere [63].

### Strengths and limitations

A strength of this study is the triangulation of data sources, which provides different perspectives on the topic. By triangulating the methods of data collection, we gained insights we would not have had if we had only carried out interviews. The first author’s experience and knowledge from palliative care can be considered a strength for the contextual understanding of HCNs facilitating of PHDs. She has formal education in palliative care and hands-on clinical experience in institutional palliative care. Nevertheless, she is not affiliated with the home care service where the study was conducted. The findings suggest that FE is a beneficial way of gaining knowledge about how HCNs facilitate PHD. Draper (2015) points out how ethnography is suitable for nursing research where practice is convergent on the experience of giving and receiving care. FE can shed light on experiences articulated by the HCN (the emic perspective) and the collective understanding of practice (the etic perspective) are how these are informed and influenced by each other [64]. By playing with both emic and etic perspectives, we gained rich insights into the field [29]. Although the first author took every available opportunity to spend time in the field with HCNs, field observations were limited in this study. However, the first author took action on every call from HCNs to spend time in the field.

To ensure rigor (trustworthiness), we practiced a reflective approach throughout the study – from developing the research questions to data collection and analyses – without compromising the richness of the data. To demonstrate transparency, an audit trail was created using a reflective diary, ethical considerations, a methodological journal, and data analysis chronologies [65]. This was carried out in addition to method triangulation, bracketing, member checking, and thick descriptions [66]. Member checking was used during and after the interviews and field visits by asking participants if the observations, notes, and assessments made by the researcher were correct and in line with the participant’s own beliefs and intentions. This method can reduce any decontextualizing or misinterpretation of the participants` descriptions in the analysis process and demonstrates veracity. We followed the COREQ guidelines for reporting qualitative research [67].

### Implications for practice

Both organizational and individual factors influence HCNs’ practices. A broad understanding of the complexity of the HCN’s role when facilitating PHD or providing palliative care in patients’ homes is important. In highlighting these challenges, we intend to inform future policies and possibly contribute to more sustainable palliative care in home care. This could potentially enable more PHDs for patients wanting to die, or spend their last days, at home. Consequently, the impact and degree of stress experienced and the coping strategies adopted by HCNs require further research. Using an ethnographic approach highlighted how HCNs may subtly discourage a PHD when they feel they don’t have the capacity to facilitate it. This finding highlights the ways that inadequate staffing of home care affects access to a PHD.

This research carries significant implications for healthcare delivery models that promote home-based care, particularly in their heavy reliance on informal caregivers. This begs the question of whether these systems are merely shifting the care responsibility onto families and how we can enhance support for these families. And if so, how do we support a person’s wish to die at home when they don’t have adequate family support? Future research should include innovative and sustainable ways of providing palliative care in the home that offers desirable outcomes for patients, NOK, and HCNs.

The Norwegian healthcare services has similarities with other European countries, and our findings may be applicable to other nations. HCNs’ facilitation of PHD has been addressed in previous studies, and related findings such as sparse resources and staffing shortages have been identified. This study adds to the evidence by demonstrating the challenges experienced by HCNs while simultaneously having positive attitudes towards PHD.

## Conclusion

Our findings reveal shortcomings in the clinical practice of facilitating PHD and identify the tensions that arise when planning a death in the context of the patient’s home. HCNs are pushing the boundaries of their practice by embracing PHDs, while at the same time compensating for a fragile system by going above and beyond for patients and their NOK. The role of NOK as the primary carer is demanding, and the recognition of their needs is crucial. NOK appear to be a requirement when facilitating a PHD, but they need individualized support and attention.

HCNs in fact take responsibility for a faulty system, and it seems that they are compensating for organizational barriers such as sparse staffing by going above and beyond their normal duties. HCNs seem to welcome and want more PHDs in order to continue evolving and to gain valuable experience. The HCNs are apparently facing a constant dilemma between the disparity between the ideal nursing standards of care and their limited capacity to meet such standards. Our findings suggest that some patients are able to die at home due to the extra effort HCNs put into their work.

The findings of this study reinforce previous research that explores the views of HCNs facilitating PHD and providing palliative care in the home. However, this study also provides observations of HCNs’ practice when they provide said palliative care and gives insights into poorly resourced home care services and how HCNs make ends meet without `*enough hands*` in a fragmented system. This warrants attention because the nurse’s role is an important topic in today’s health care climate. The challenges in recruiting and retaining nurses in the profession are well-known, particularly within primary health care. This paper highlights some of the challenges HCNs face when doing everything they can within their capacity to give patients and their NOK a good death at home.

### Electronic supplementary material

Below is the link to the electronic supplementary material.

Supplementary Material 1

## Data Availability

Data are available from the authors upon reasonable request.

## Declarations

This study was reported to the Regional Committee for Medical and Health Research Ethics in Northern Norway (REC). An exemption from the duty of confidentiality was obtained from REC (2019/605). This study was performed according to Norwegian Centre for Research Data (NSD) (77356) regulations and in line with the Helsinki declarations [68]. All participants were informed verbally and were provided with a participant information sheet and gave their written informed consent to participate in interviews and observations.
